# Impact Analysis of Temperature and Humidity Conditions on Electrochemical Sensor Response in Ambient Air Quality Monitoring

**DOI:** 10.3390/s18020059

**Published:** 2018-01-23

**Authors:** Peng Wei, Zhi Ning, Sheng Ye, Li Sun, Fenhuan Yang, Ka Chun Wong, Dane Westerdahl, Peter K. K. Louie

**Affiliations:** 1 School of Energy and Environment, City University of Hong Kong, Tat Avenue, Kowloon, Hong Kong, China; wp5621679@gmail.com (P.W.); ttllttttlltt@gmail.com (S.Y.); sunliapply@126.com (L.S.); fhyang2012@gmail.com (F.Y.); kcwong.joe@gmail.com (K.C.W.); danewest03@gmail.com (D.W.); 2 Environmental Protection Department, the Government of the Hong Kong Special Administration Region, 33/F Revenue Tower, 5 Gloucester Road, Wan Chai, Hong Kong, China; plouie@epd.gov.hk

**Keywords:** electrochemical sensor, correction method, urban air pollution, low cost sensors

## Abstract

The increasing applications of low-cost air sensors promises more convenient and cost-effective systems for air monitoring in many places and under many conditions. However, the data quality from such systems has not been fully characterized and may not meet user expectations in research and regulatory uses, or for use in citizen science. In our study, electrochemical sensors (Alphasense B4 series) for carbon monoxide (CO), nitric oxide (NO), nitrogen dioxide (NO_2_), and oxidants (O_x_) were evaluated under controlled laboratory conditions to identify the influencing factors and quantify their relation with sensor outputs. Based on the laboratory tests, we developed different correction methods to compensate for the impact of ambient conditions. Further, the sensors were assembled into a monitoring system and tested in ambient conditions in Hong Kong side-by-side with regulatory reference monitors, and data from these tests were used to evaluate the performance of the models, to refine them, and validate their applicability in variable ambient conditions in the field. The more comprehensive correction models demonstrated enhanced performance when compared with uncorrected data. One over-arching observation of this study is that the low-cost sensors may promise excellent sensitivity and performance, but it is essential for users to understand and account for several key factors that may strongly affect the nature of sensor data. In this paper, we also evaluated factors of multi-month stability, temperature, and humidity, and considered the interaction of oxidant gases NO_2_ and ozone on a newly introduced oxidant sensor.

## 1. Introduction

Air pollution is widely recognized as one of the main causes of adverse health effects, with documented association to acute and chronic diseases. Important components of polluted atmospheres include particle and gas phase pollutants, such as nitrogen dioxide (NO_2_), ozone (O_3_), and carbon monoxide (CO) [1,2]. They are either directly emitted from primary pollutant sources such as vehicular traffic, ships, power production etc., or formed in the atmosphere during photochemical processes [3,4,5].

Effective air monitoring is essential in better understanding the sources and nature of air pollution for exposure assessment, environmental policy formulation, and evaluation of the effectiveness of such policies [6,7]. While regulatory monitoring stations provide detailed and accurate air pollution measurements, the instruments are expensive and bulky, and require conditioned housing, considerable maintenance, and on-site calibration [8]. The existing air monitoring systems sited in urban locations are often limited in number and have a primary objective of establishing compliance with air standards and guidelines [9]. Further, traditional monitoring stations are generally located away from source emissions and may not allow consideration of the impacts of local sources on ambient air quality.

With the increasing needs of air quality data for scientific purposes, alternative air monitoring approaches have become trendy with promises of lower cost and power consumption, high temporal resolution, flexibility, and convenience in operation [10,11]. There is a growing body of literature documenting the performance and application of compact, low-cost, and portable gas sensors [12,13,14,15,16]. These sensors operate by different working principles [17]. For example, photo-ionization based sensors are often used for detecting volatile organic compounds (VOC) [18]; optical sensors are commonly used for measuring gas absorption at a specific infrared wavelength window that is sensitive to carbon dioxide (CO_2_) and methane (CH_4_) gas [19]; and sensors using semi conductive metal oxides which exhibit changes in conductivity when exposed to gas molecules [20]. Electrochemical sensors are widely employed to detect several of the common gas pollutants that can be reduced or oxidized on electrodes resulting in linear response in current [21,22].

Electrochemical cell based sensor technology offers a variety of advantages including linear response to concentration, low cost in fabrication, relative fast response, light-weight, and low power consumption, all of which are desirable for the so-called “next generation” air monitoring. A few large scale ambient air monitoring programs have employed electrochemical sensor based technologies, such as the EuNetAir project which have been deployed for air quality measurement and modeling at an international level [23,24], and the Cambridge University network that provided continuous measurement of CO, NO, NO_2_, and environment parameters with high temporal resolution for quantification of human exposure [21,25]. Mobile sensor platforms have shown the ability of monitoring city scale variation, such as Citi-Sense-MOB [26]. A multi-gas sensor system was used for a study of emissions from volcanos, taking advantage of the fast response of electrochemical sensors [27,28]. Sensor networks of high spatial density can also be used for quantifying source attribution, which distinguish pollutant contributions from local background emissions to local and non-local receptors [25]. Sensor based monitoring can also measure personal exposures when assembled into small battery powered systems that are carried by people as they go about their normal activities.

Sensor based monitoring applications bring new opportunities for air monitoring, however, important issues exist regarding data quality in sensor applications. Studies have demonstrated that sensor data are subjected to considerable influence from environmental factors such as temperature, humidity, and even interference of other air pollutants [14,15,21]. Considerable efforts have been made to understand these factors, with varying degrees of complexity and success, including machine learning methods for correcting raw data. Since application of sensors into real-world environments requires the deployment outside the laboratory and direct exposure to complex and dynamic ambient conditions, it is crucial to document the sensor performance and compensate the ambient factors under various these real-world conditions. These challenges include: 1. sensor responses to a wide range of ambient environmental conditions; 2. measurement of target gases over wide ranges of concentration, 3. sensor cross-sensitivity to multiple gases; 4. conversion of the sensor electrical output responses to the real concentration; and 5. lack of well proven protocols for quality assurance and control for sensor field application.

Acknowledging these issues, we designed experiments to quantify the impact of two important environmental factors (i.e., temperature and humidity) on the sensor response for criteria gas pollutants. An experiment-based method of sensor calibration was used to convert the sensor output to the gas concentration, and different methods were tested to correct the environmental impact on sensor response and improve the sensor data quality. Multi-month assessments were made to consider the stability of senor response. Finally, we carried out detailed error analysis on the corrective models in the real-world application.

## 2. Experimental Methodology 

The working principle of an electrochemical sensor is that the target gas reacts with the sensor cell that contains an electrolyte and either two or three electrodes. The “working” electrode generates electric current while oxidized or reduced by the target gas; a “counter” electrode is used to balance the reaction of the working electrode and generate equivalent current which is proportional to the target gas. Some sensors include a “reference” electrode to anchor the working electrode and helps maintain constant sensitivity, good linearity, and enhanced sensitivity for the target gas [29]. Laboratory and field experiments were conducted to evaluate the characteristics of sensor response to a range of gas concentrations and environmental factors. 

### 2.1. Laboratory Tests

**Figure 1:**
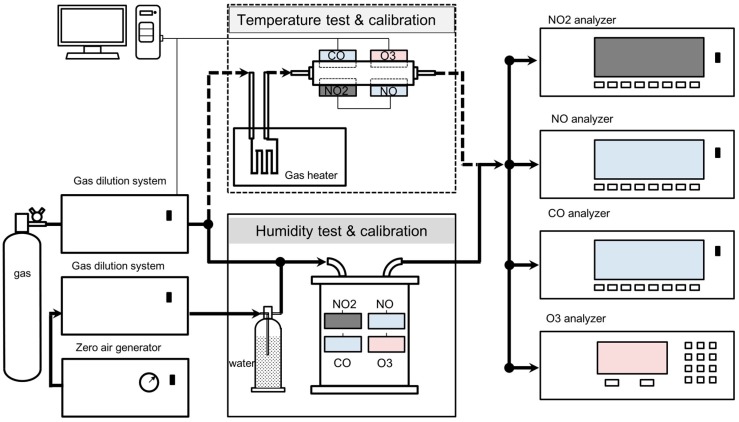
Laboratory setup for sensor linearity, cross sensitivity, and zero air drift test.

Laboratory tests were conducted to establish the relationship of output from selected electrochemical sensors with concentration of individual gases (NO_2_, NO, O_3_, CO) under controlled laboratory conditions. Four models of commonly used electrochemical sensors including NO_2_-B42F (NO_2_), NO-B4 (NO), CO-B4 (CO), and O_X_-B421 (combined oxidant gases NO_2_ and O_3_) (Alphasense, UK) were tested to evaluate the sensor linearity, the impact of temperature and humidity, cross interference, and for multi-month response stability (drift). Ozone values were derived by subtracting the observations of the NO_2_ sensor from that made by the oxidant sensor. Prior to the laboratory evaluation, multiple sensors from the same batch of manufacturer shipment were tested to check their internal consistency. There was good agreement of their linearity in response to gas concentrations, as reported in literature. The detailed laboratory tests and field evaluation reported the representative data from each type of gas sensors. The test setup is composed of 4 major components, as shown in Figure 1.

#### 2.1.1. Standard Gas Generation

The target gases were produced by two methods depending on the gas of interest. CO and NO concentrations were produced by diluting gas of known concentration from standard gas cylinders (with documented content within certification validity periods) with pollutant-free air produced by a zero air generator T701H (Teledyne-API, San Diego, CA, USA). The target gas concentration and zero air flow rate controlled by a Lotun Science S102D dilution system (Lotun, Taiwan). The flow rate and time of each step were controlled by a PC. Ozone and NO_2_ were produced by reacting NO from a cylinder by the T700U (Teledyne-API, San Diego, CA, USA) dynamic dilution calibrator. It includes three mass flow controllers and is capable of producing NO_2_ and ozone calibrations down to 3 ppb. Flows were confirmed by regular comparisons with a Gilibrator 2 (Sensidyne, St Petersburg, FL, USA) flow meter which was factory calibrated 6 months prior to use. 

#### 2.1.2. Sensor Test Apparatus

The 4 gas sensors were fixed to an air tight Teflon manifold and sample air was passed through the manifold with a flow rate of 1 L per minute (l pm). All the connections were made of Teflon tubing and stainless steel connectors to minimize gas losses. For linearity tests, the gases of different concentrations were passed directly through the sensor manifold at a stable temperature of 22 ± 0.5 degrees. 

To test the effects of temperature and humidity conditions on the sensor performance, temperature and relative humidity were controlled, as shown in the middle panel of Figure 1. The upper component is a temperature controller, in which the gas stream passed through a long coiled tube that was heated or cooled by a liquid bath using VWR Signature Circulating Bath (Model 1146D). The target temperatures ranged from 15 to 34 °C, conditions typically encountered in the urban atmosphere in Hong Kong. 

In humidity tests, the sensors were installed inside a glass chamber and the target gas humidity was controlled by mixing the standard gas with zero air that was wetted by passing through glass water bubbler, as shown in Figure 1. The gas mixture concentration and humidity are determined by the flow rate ratio of the two streams. 

#### 2.1.3. Reference Gas Instruments

NO_2_ analyzer (Teledyne-API T500-U, San Diego, CA, USA) and NO&NO_2_ analyzer (Teledyne-API-T200UP, San Diego, CA, USA), CO analyzer (Teledyne-API-T300U, San Diego, CA, USA) and an Ozone analyzer (Ecotech-Serinus10, Knoxfield, Victoria, Australia) were operated in this study and served to produce “reference” grade data for laboratory calibration studies. The U series NO_x_ and CO analyzers are trace gas level monitors, while the Serinus is widely used in ambient air monitoring. The performance of each of these monitors was determined by on-site comparison with validated regulatory monitoring data. The manifold and chamber were connected to analyzers with Teflon tubing. 

#### 2.1.4. Data Collection 

Sensors output voltages (both reference and active from the electrochemical sensor) and humidity and temperature data were logged by an Arduino MCU board (Mega ADK, Scarmagno, Italy) and transmitted to the PC every 5 s.

Prior to the tests, each sensor was powered on for at least 48 h stabilize the output [10,30]. For each sensor calibration and performance test, zero air was pumped through the Teflon manifold to purge tubes and chambers for 15 min. Linearity tests were carried out in a stable indoor environment with temperature remaining stable at 22 °C and relative humidity at 40%. NO, NO_2_, and oxidant sensors were tested at ppb levels. NO, NO_2_, and ozone concentrations steps were set at 5, 50, 100, 150, and 250 ppb, while CO concentration tested included 0.1, 0.5, 1, 1.5, and 2 ppm. Each step ran for 15 min with the data from the first 2 min removed to ensure of adequate response time and stabilization of the test system. After linearity test, the sensors cross interference was also tested when pumped into one specific target gas.

It is well established that variations in ambient conditions of temperature and relative humidity have significant impacts on sensor response to the pollutant concentrations [31,32]. To address this, tests were also performed under combinations of temperature and humidity settings. For each sensor, a target gas of different concentrations including zero air was pumped into the manifold with humidity of 17%, 30%, and 48% for each step cycle and temperature of 16, 19, 33, and 36 °C. 

Electrochemical sensors are known to produce linear response with target gas concentrations in repeated laboratory tests, however, they may operate for weeks to months in a variety of ambient conditions with little attention by users to continued data accuracy. A number of studies have shown a drift of sensor sensitivity and output to the gas pollutants over long term deployment [33]. In this study, we evaluated the stability of sensor response over a period of two months. The multiple-sensor manifold was supplied with zero air at a stable temperature (22 °C) and relative humidity (40%) each day with a 1-h duration, while for the remainder of the day, the sensor was powered at all times with outdoor ambient air passing through the manifold. During the test period, the ambient air temperature ranged from 17 °C to 24 °C and relative humidity ranged from 54% to 95%.

### 2.2. Field Tests

Field evaluation tests were carried out from 14th through 25th of February 2015 at the Hong Kong Environmental Protection Department (HKEPD) roadside Air Quality Monitoring Station (AQMS). The station is located at the roadside of the junction of Charter and Des Voeux Roads in Central, Hong Kong (Latitude: 22.28197, Longitude: 114.15758). Both roads are busy with traffic in the heart of the business district. The AQMS is equipped with regulatory grade gas analyzers that report data on CO, O_3_, SO_2_, NO, and NO_2_ on an hourly basis. HKEPD provided raw data from these analyzers at 1-min resolution for our use in assessing sensor platform operation. These data were averaged to and compared with sensor data at 5-min resolution.

Three identical sensor platforms equipped with the NO, NO_2_, and CO, and oxidant sensors were placed on top of the AQMS station rooftop with a distance of 1 to 2 m from the inlets of reference equipment. An Arduino-based MCU served as an integration module for data communication. Power for each was provided by a 24 V, 20 Ah Lithium Ion battery. Data were transmitted to the server in the laboratory via GPRS using an interface card added to the MCU. A detailed description of this system is presented in the work of 2015 Hong Kong Marathon sensor network monitoring [34].

### 2.3. Correction Model Development

Electrochemical sensors have two raw outputs, including active (V_Act_) voltage from working electrode and reference (V_Ref_) voltage from auxiliary electrode. The Act voltage responds to target gas concentration directly and is also affected by environmental parameters, while the Ref voltage serves to anchor the working electrode voltage with response only to the change of environmental parameters. The difference (V_Diff_ = V_Act_ − V_Ref_) of the Act and Ref voltages is proportional to the target gas concentration when measured in the stable environment. 

The equation to express this proportionality is presented in Equation (1) as Model 0,

Model 0: Conc = S × V_Diff_ + B + D (1)

where V_Diff_ is in mV, the coefficient S represents sensor response sensitivity in ppm/mA or ppb/nA, and B is the baseline response in concentration, while D is the correction for drift and the cross interference from other gases [33]. In Equation (1), S and B can be obtained by multiple-point calibration between sensor output and target gas concentration.

For practical application, variations in temperature and relative humidity have been shown to impact sensor output. Based on the test results from this study, S and B in Equation (1) can be generalized by the following simple linear equations, as illustrated in Figure 3:

S_RH,T_ = a_1_ × RH + a_2_ × T + a_3_ (2)

B_RH,T_ = b_1_ × RH + b_2_ × T + b_3_ (3)

If the impact of temperature and relative humidity is considered, Model 0 may be further transformed to Model 1:

Model 1: Conc = (a_1_ × RH + a_2_×T + a_3_) × V_Diff_ + b_1_ × RH + b_2_ × T + b_3_ (4)

Further, while comparing the sensor response to the varying conditions, different sensor reference electrode outputs had different degrees of response to temperature and humidity. We determined the regression of the V_Ref_ with T and RH. Table 1 lists R^2^ of the regression and slope coefficients of T and RH on V_Ref_. There is a statistically significant correlation of NO sensor reference voltage (R^2^ = 0.94, *p* < 0.001), which is dominated by temperature with high T-weight of 3.16 versus very small RH-weight of 0.03 in magnitude, thus the impact of RH can be neglected in this case. The NO_2_ sensor reference voltage was found to demonstrate a second order relationship with ambient RH, but such correlation is much lower at R^2^ = 0.56, while for CO and O_3_, there is no significant correlation, with R^2^ = 0.35 and 0.45, respectively.

Based on these results, assuming a linear relation between V_Act_ and V_Ref_ to correct working electrode voltage response to gas concentration may not be optimally considered by direct subtraction; and separate regression of V_Act_ and V_Ref_ seem advantageous. The model can be transformed into the following possible forms:

Model 2: Conc = (a_1_ × RH + a_2_ × T + a_3_) × V_Act_ + b_1_ × RH + b_2_ × T + b_3_ (5)

Model 3: Conc = (a_1_ × RH + a_2_ × T + a_3_) × V_Act_ + (b_1_ × RH + b_2_ × T + b_3_) × V_Ref_ + b_4_ (6)

In Equation (5), we assumed V_Act_ directly responds to the concentration and V_Ref_ is not used for correction, while T and RH are included as independent coefficients. In Equation (6), V_Act_ and V_Ref_ are both employed independently in which V_Act_ is assumed to directly respond to target gas, while V_Ref_ is included to reflect impact of T and RH. Separate T and RH correction is removed to avoid over correction. The data analysis was based on Python 2.7 and Origin software.

## 3. Results and Discussion

### 3.1. Laboratory Evaluations

#### 3.1.1. Linearity Test under Stable and Variable Conditions

**Figure 2:**
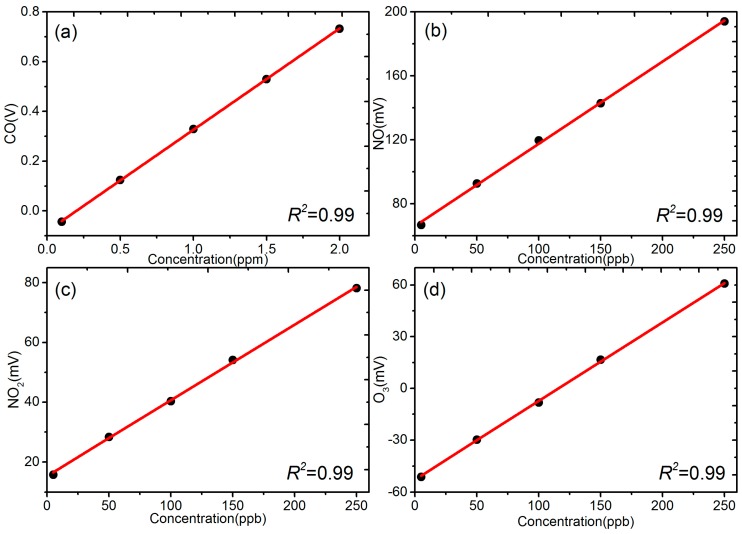
Laboratory multiple point linearity test with standard gas at 22 °C and 40% (**a**) CO (carbon monoxide), (**b**) NO (nitric oxide), (**c**) NO_2_ (nitrogen dioxide), (**d**) O_3_ (oxidants).

Figure 2 shows the correlation between raw outputs voltage from sensors and standard gas concentration measured using regulatory grade air monitors that served to produce a reference value for the pollutants of concern. Each data point represents the average of 10 min values for each concentration step. As expected, the sensors demonstrated high linearity with specific gas, and R^2^ values are higher than 0.99 for all 4 pollutants, consistent with our prior studies and those reported by others [17,21,34,35]. However, the linearity response of sensors was determined once stable environmental conditions were established after sensor “stabilization”. This demonstrates that the electrochemical sensors perform well under ideal conditions. 

**Figure 3:**
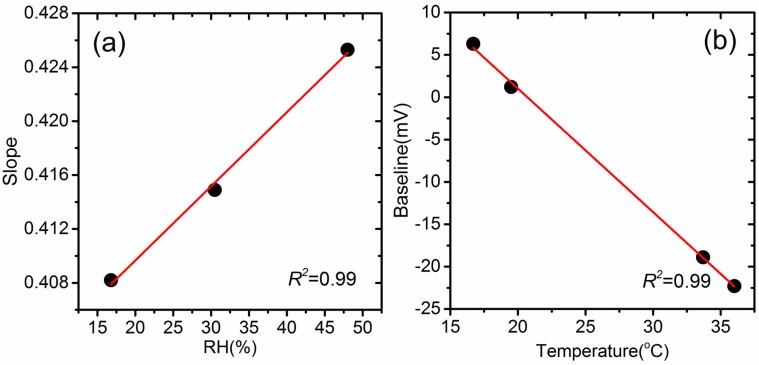
CO sensor response to the change of ambient factor (**a**) RH dependent slope of output and concentration (**b**) Temperature dependent baseline voltage.

In order to establish the relation between the ambient parameters (RH and temperature) and the voltage output of sensors, the slope and intercept produced from the regression of each linear test under each temperature or humidity condition were plotted in Figure 3, taking CO as an example. The other sensors showed a similar linear relation but with different slopes and intercepts. The left panel shows a positive and linear correlation between the sensor response slope and relative humidity, while the right panel shows a negative relation of the temperature and baseline voltage while passing zero air through sensors. The change of slope with increasing RH from 15% to 48% was around 5%, and baseline voltage shifted from 7 mV to −22 mV when temperature increased from 18 °C to 36 °C, equivalent to a drift of CO concentration of about 0.1 ppm, according to Figure 2a. The protocol for testing the influence of temperature and relative humidity did not include highly humid conditions because our system was unable to produce these levels. However, the ambient factor dependent sensor response clearly indicates the need to include the correction of the S (slope) and B (baseline) as shown in Equation (1), especially for sensor applications in ambient monitoring with strong diurnal and seasonal cycles of climate conditions. The detailed correction method is shown in Section 3.2. Further testing under more extreme humidity conditions requires protocols that are under development.

These laboratory tests provide a basis to determine the limits of detection (LOD) for each sensor. The standard deviation for each sensor was estimated under zero air conditions after calibration with reference machines. The LODs can be estimated as 3X of standard deviation [21,35,36].

#### 3.1.2. Long Term Drift

**Figure 4:**
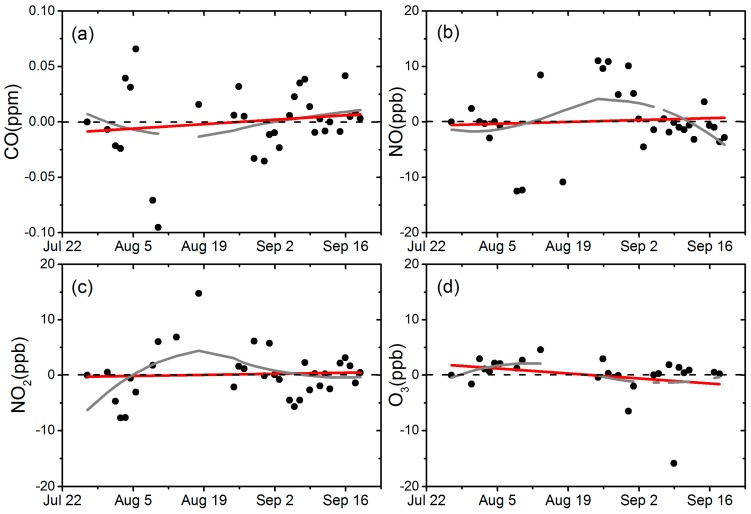
Zero air test for sensor drift evaluation for CO (**a**), NO (**b**), NO_2_ (**c**), O_3_ (**d**), a linear fitting (red line) and loess smoother (gray line) was added.

Figure 4 presents the performance of the sensors during the 2-month drift test. Each data point represents the calculated pollutant concentration according to Equation (1) from the measured sensor voltage output (V), with the slope (S) and baseline (B) values acquired from regression for each gas under stable conditions. The first data point is the observed initial zero concentration, with D initially set as 0 for reference.

There are two important observations from the test results: 1. during the test period, no consistent D values were found and they scattered within a relatively narrow concentration band with no clear pattern. The Minimum-Maximum of the concentration output for NO_2_, CO, NO, and O_x_ sensors from this test were −7.7 to 14.8 ppb, −0.10 to 0.07 ppm, −12.5 to 11 ppb, and −15.8 to 4.6 ppb, respectively, shown in Figure 4. Note the test conditions for the zero air with stable and consistent temperature and humidity, which eliminated the possibilities of variation in B due to the change of sensor conditions. The variation in the sensor output may be due to the inherent electronic noise or the physical change of the sensor itself that affected the sensors’ response after exposure to variable ambient conditions and 2. In addition, a linear fitting was performed with measurements shown as the red line in Figure 4. For NO, NO_2_, and O_3_ values, the overall average drift is <2 ppb per month and <0.02 ppm per month for CO sensor. Meanwhile, a loess smoother was presented which showed non-uniform and non-significant trend for 4 sensors. Although only two months of continuous measurements were carried out, the accumulated drift may be high for the longer term and multi-month sensor operations. This strongly suggests the necessity of periodic calibrations or other procedures to account for sensor drift performance, even for high concentration range measurement. Further studies are underway to evaluate methods to reduce the impacts of long term drift in these sensors.

#### 3.1.3. Cross Interference

The cross interference of sensor response to non-target gases was also evaluated. Table 2 shows the results of the relationship between the 4 sensors and gases. The data represents the ratio of sensor response in calculated concentration to non-target input gas concentration calculated in percentage. CO, NO, and NO_2_ sensors showed no evidence of cross interference when exposed to other gases, consistent with that claimed by the sensor vendor specifications [37]. The O_x_ sensor (O_x_-B421), however, had a nearly 1:1 linear response to pure NO_2_ gas, as reported in other studies and the sensor specification [21]. The NO_2_ sensors investigated in this study had a filter membrane that removes ozone before it reacts with cell electrolyte. This allows NO_2_ sensor to report NO_2_ only in ambient air that contains ozone. O_3_ has been shown to produce positive artefactual readings on some NO_2_ electrochemical cells without this filter [38]. The correction method for O_3_ thus needs to take into consideration the co-existing NO_2_ gas, by subtracting the calculated O_x_ (O_3_ + NO_2_) with measured NO_2_ concentration, given the 100% response ratio shown in Table 2. All O3 data presented in the following sections are calculated by subtracting NO_2_ concentration. This finding of NO_2_ gas and O_x_ sensor interference is not unexpected, but it has not been quantitatively documented [21]. The inclusion of this ozone filter added to the NO_2_ sensor combined creates new possibilities for accurate measurement of O_3_ and NO_2_ with electrochemical cells. Care should be taken when viewing the results of prior studies, performed before 2015, due to this sensor design factor for Alphasense NO_2_ sensors. For example, research findings presented by Spinelle et al. [14] made use of sensors that did not include ozone filters on Alphasense NO_2_ sensors, and did not include subtraction of observed NO_2_ values from an oxidant sensor to determine ozone concentrations. Sensor cells for NO_2_ employed in other field and laboratory studies may or may not have included this filter. The presence of this filter feature is uncertain on sensors produced by other sensor vendors or in monitoring systems that report NO_2_ from electrochemical sensors.

### 3.2. Evaluation of the Correction Models

**Figure 5:**
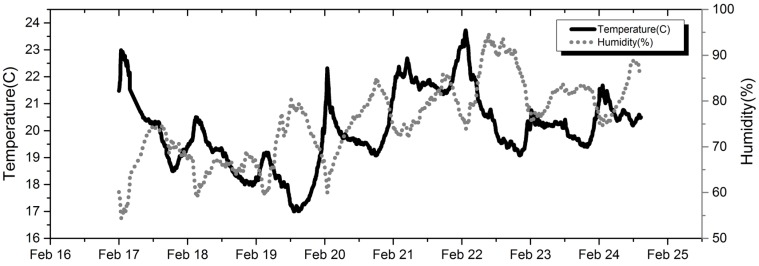
The ambient temperature and relative humidity during field test.

Figure 5 shows the time series of ambient conditions during the field test carried out at the urban site. There was a large variation of the relative humidity (54% to 95%) and mild range of temperature (17 to 24 °C) during the period, which produced a good opportunity for the evaluation of different models under Hong Kong conditions. It should be noted although the test period is relatively short, lasting 8 days, it serves to compare the performance of different correction models under varying conditions. Longer time series of data is preferred if one were to evaluate the long term drift of sensor response in the field, which should be the scope of future studies.

To evaluate the performance of three models, the field measurement data were separated into two sets. One model from 17th to 21st February 2015 was used to perform the multiple linear regression, illustrated in Section 3.2, with the AQMS gas data which was provided with 1-min resolution to obtain the coefficients of different models. A total of about 1,100 data points were used for the regression from the valid 5-min average sensor and AQMS data. Further, the other experimental data was collected from 22nd to 25th February 2015 to evaluate the validity and performance of model in predicting ambient gas concentration. For CO, NO, and NO_2_ gases, the concentrations were calculated directly from the proposed models, while O_3_ concentration was determined prior to statistical analysis.

**Figure 6:**
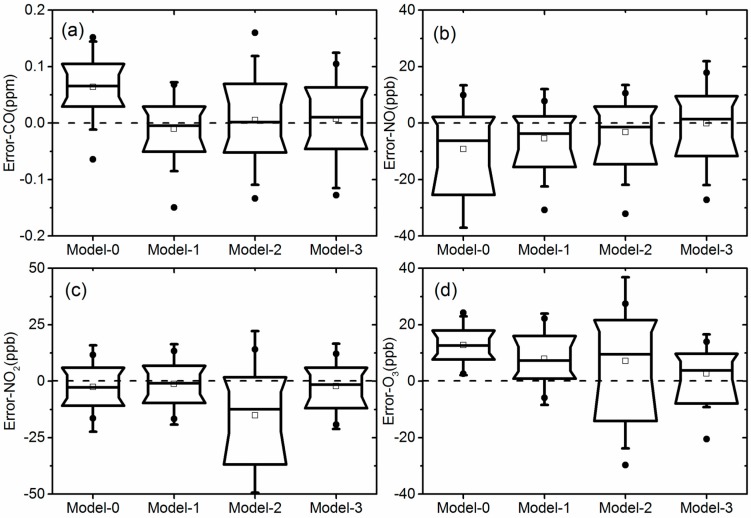
Box plots of error distribution of 4 models: (**a**) CO, (**b**) NO, (**c**) NO_2_, (**d**) O_3_.

Table 3 shows the statistics of the regressions between sensor and reference instrument data using the first three days of measurement data as calibration. The difference or error between the calculated sensor concentration and the reference data in the 2nd time period was further analyzed as validation. R^2^ is the correlation coefficient of the regression. “1σ” is one standard deviation of errors to represent the spread of error distribution and “Mean” is the average of errors to represent the accuracy of the model performance. Figure 6 further illustrates the box plots of error distribution for all the models, using data from the validation period. The solid round and rectangular dots represent the 1% and 99% percentile, and mean value, respectively. The 4 inflection points of the box margin from the bottom to top represent 10%, 25%, 75%, and 90% percentile of the errors, and the bar in the middle of the box is the median value.

In general, the introduction of T and RH correction from the use of the 3 different models showed clear improvement of the sensor performance, compared with Model 0. For CO and NO_2_ gases, Model 1 had a better performance for all 3 criterions in Table 2, and also showed a narrower spread of errors in the 25 and 75 percentile band in Figure 6. The direct usage of V_Diff_ seems to correct the impact of variation of ambient condition by subtracting V_Ref_ from V_Act_. Model 3 of NO and O_3_ gases performed well in terms of mean error with similar R^2^ and standard deviations, compared to the other two models. Model 3 introduced V_Ref_ as an independent parameter for T and RH correction and used V_Act_ as direct input of sensor response to pollution concentration. The good performance for NO sensor agrees with the results in Table 2, showing a high degree of correlation between NO V_Ref_ with T and RH independently. For O_3_, the result may be complicated by the fact that NO_2_ was subtracted from the O_x_ sensor output result. Thus, inaccuracies in the calculated ozone values are contributed by any inaccuracies in both NO_2_ and oxidant sensor response. Further studies will consider the nature and contribution of factors that influence the NO_2_ values calculated from these sensors.

### 3.3. Error Analysis

**Figure 7:**
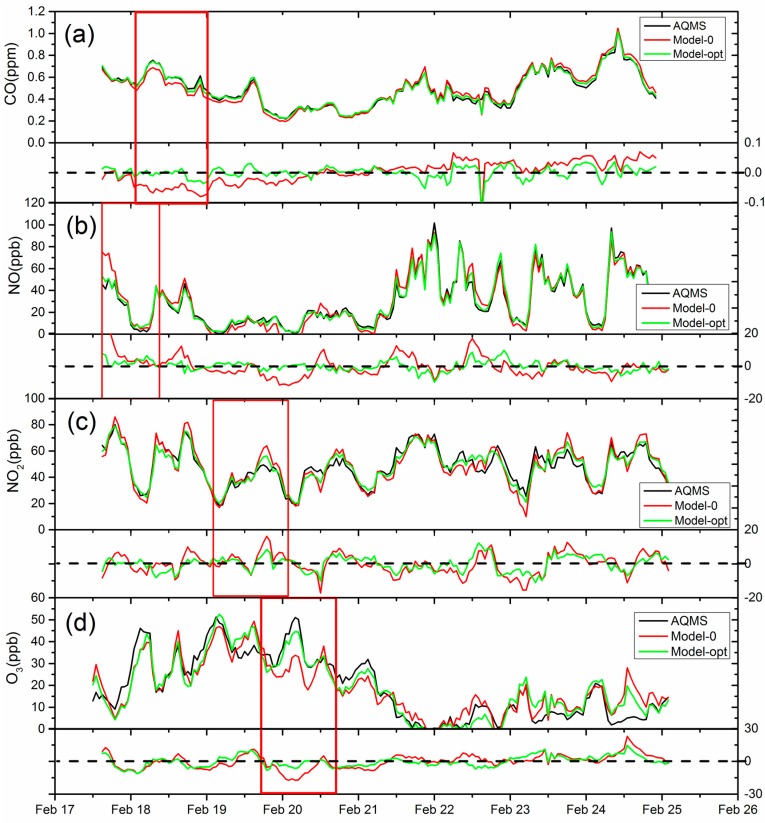
The comparison of hourly AQMS (Air Quality Monitoring Station) and sensor data (upper panel) and the errors of uncorrected Model 0 and optimal Model (lower panel) (**a**) CO, (**b**) NO, (**c**) NO_2_, (**d**) O_3_. The red rectangle represented the largest deviation period of the 2 models compared.

Figure 7 shows the time series of hourly AQMS concentration of pollutants compared with the sensor data derived from Model 0 (without T and RH correction) and the optimal Model (Model-opt) chosen from the regression analysis, i.e., Model 1 for CO and NO_2_, and Model 3 for NO and O_3_. The comparison of errors between calculated and measured concentration for the two models is shown in the lower panel of each subplot. The data used here covered the field test period of 8 days to validate the effectiveness of the correction models in replicating desired performance. In the rectangular box that highlights the period with significant difference in Figure 7, the deviation of uncorrected Model 0 data from AQMS data could reach as much as 15 ppb for NO_2_ and NO, and 20 ppb for O_3_. Model 0 for CO performed reasonably well, as both models tracked each other. The T/RH corrected model for all the sensors demonstrated a greater agreement with the AQMS data than the uncorrected Model 0, especially for those periods when Model 0 has a large deviation, clearly demonstrating the importance of ambient condition correction for these sensors.

**Figure 8:**
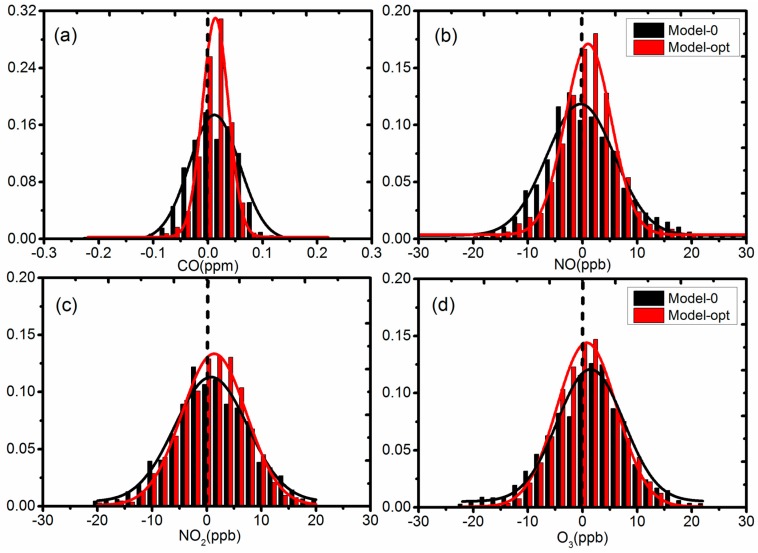
Histogram of errors from Model 0 and optimal Model fitted with normal distribution curves (**a**) CO, (**b**) NO, (**c**) NO_2_, (**d**) O_3_.

Figure 8 presents the histogram of the errors from Model 0 without correction and optimal model chosen from regression analysis. The errors are calculated from the 5-min resolution AQMS and sensor data, and they closely follow a normal distribution with mean value around 0 and long tails along both sides. The red and black lines in the figure are the fitted normal distribution curves for Model 0 and optimal model, respectively. As shown in the figure, the errors from the optimal model clearly had narrower distributions. F-tests were performed for all four sets of sensor data and there was a significant difference between the variation of errors from Model 0 and the optimal model (*p* < 0.001) at a significance level of 0.05, demonstrating the improvement of measurement precision using the optimal model. Using 1 standard deviation of the error distribution as an indicator, the CO, NO, NO_2_, and O_3_ results showed an improvement of 41% from 8.3 to 5.9 ppb, 35% from 0.05 to 0.03 ppm, 22% from 7.4 to 6.1 ppb, and 32% from 7.4 to 5.6 ppb, respectively.

**Figure 9:**
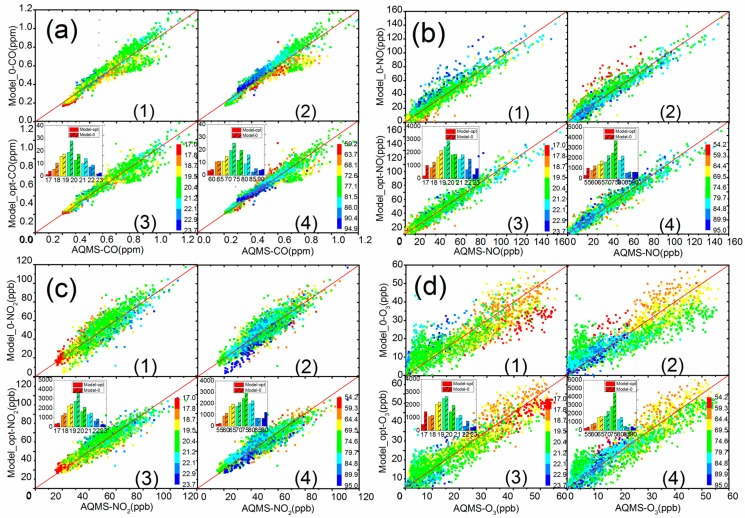
Scatter plot of AQMS and sensor data by Model 0 and corrective Model-opt. (**a**) CO, (**b**) NO, (**c**) NO_2_, (**d**) O_3_. Insets represent the cumulative errors in each temperature and relative humidity bin. Subplots 1 and 3 are color categorized plots by temperature for Model 0 and Model-opt, respectively. Subplots 2 and 4 are color categorized plots by relative humidity for Model 0 and Model-opt, respectively.

Figure 9 shows the scatter plots between the AQMS reference data with the sensor data from uncorrected (Model 0) and corrected (optimal model) models. Each data point in the scatter plot is also color coded to indicate the corresponding ambient conditions of T and RH. A 1:1 line is shown in the plots for reference. The cumulative errors of the sensor data from two models are plotted as a bar chart in the subplot. T and RH were equally separated into 8 bins according to the range of measured data and the bar for each bin represents the summation of the errors within the bin.

For CO, there exist larger errors in low to middle T range (bins from 17.0 °C to 20.4 °C) and medium RH range (bins from 77.1% to 86.0%) in uncorrected Model 0, where there is a major deviation below 1:1 line, as shown in the scatter plot. This means a remarkable underestimation of pollutant concentration from sensor data in this T and RH range. The introduction of the corrective Model 3 improves the performance with less scattering sensor data from AQMS data. Taking the ratio of accumulated errors in the T or RH bin using Model-opt model and Model 0 as an indication of improvement of sensor accuracy, the corrective Model 3 produced the accumulative error ratio of 0.31 and 0.67 in the abovementioned T and RH bins. This is equivalent to a 69% and 33% of improvement in sensor measurement accuracy.

For NO, the error distribution shows a different pattern compared with CO data. The data differing most from the 1:1 line seem to be predominately driven by the combination of high T and low RH. After application of corrective Model 1 for T and RH, the scatter plots show a more concentrated pattern along the 1:1 line with less deviation, which demonstrates the effectiveness of the correction model in reducing the sensor measurement error. The reduction is also seen in the bar charts, in which the accumulative error ratio is 0.3 and 0.31 for T bin from 22.1 °C to 23.7 °C, and RH bin from 54.2% to 59.3%, respectively.

For NO_2_, the sensor data has an overall good agreement along the 1:1 reference line, except the tail towards the low concentration range below 20 ppb from sensor Model 0 reading. These seem to be linked with combined high RH and low T conditions, as was seen for NO sensor performance. After application of the corrective Model 1, the cluster of deviated data in Model 0 is effectively removed and a much better agreement between sensor with AQMS data is demonstrated. The accumulated error ratio is 0.75 and 0.57 for the corresponding bin of T from 19.5 °C to 20.4 °C, and RH from 89.9% to 95%, respectively.

For O_3_, there seems be a wider distribution of data compared with the other three sensors. This is possibly because both NO_2_ and O_x_ sensors participated in the model calculation as described in Section 3.3, propagating the errors when estimating O_3_ concentration from sensor data. As shown in Figure 9d, low T affects the sensor performance and drives deviation of calculated O_3_ in Model 0. However, the NO_2_ sensor data perform well in the same T bins, indicating the O_x_ sensor itself may be causing larger errors at low T range. The comparison of Model 0 and Model 3 data shows the T and RH correction can substantially improve sensor data accuracy, and the deviation of data in the low T is also improved.

Overall, the error analysis of sensor performance shows sensors for each pollutant have different characteristics and responses to the change of ambient conditions. Larger errors seem to be mostly driven by the lower or higher end of the T or RH. By applying the corrective models derived from regression including T and RH factors, the sensor performance may be substantially improved.

## 4. Conclusions

This study evaluated CO, NO, NO_2_, and O_x_ electrochemical sensors performance under laboratory conditions, confirming their excellent linear response to target gas under stable conditions with R^2^ of 0.99. The sensors also show high precision at low concentration level conditions. However, temperature and relative humidity had variable impacts on the sensor response in both working electrode (active) and reference electrode voltages. For the NO sensor, a good linear relationship of temperature and relative humidity with reference voltage was found, while the three other sensors had less influence from external factors. We proposed and tested three corrective models to compensate for the impact of temperature and relatively humidity by: 1. introducing sensor output as a subtraction of active and reference voltage; 2. singling out active voltage only; and 3. separating the outputs from active and reference voltages as independent parameter inputs. Field evaluation of the three models showed Model 1 was more suitable when reference voltage responds linearly to ambient parameters, while Model 3 had good performance when reference voltage had a poor relationship with ambient parameters. Finally, a quantitative error analysis was presented between uncorrected and corrected models to identify the possible causes of the deviation of sensors with AQMS data, and evaluate the performance of proposed models in correcting the deviations. 

We have demonstrated that low-cost electrochemical sensors have high potential for use in the special purpose of ambient air quality monitoring applications in terms of their accuracy, compared to the conventional reference instruments. However, direct usage of sensor output data assuming linear relation with target gas concentrations will yield substantial errors due to the impact of ambient conditions and cross interference of gases. Careful data analysis and correction protocols are essential to guarantee good data quality. These corrective protocols seem promising in improving the sensor data performance under a range of temperature and humidity conditions, as demonstrated in this study. Further, it is clear that this added environmental data should be collected at the sensor platform for ease of use and local accuracy. These findings also provide caution to the emerging use of low-cost sensors. There is a great deal of work needed to generate reliable data from such sensors. The vendors of the cells or of monitoring systems must inform and assist the users in making these adjustments. There also remains a compelling need for further investigation on sensors, including their applicability in extreme ambient conditions and for long term drift over periods of perhaps up to a year, in order to better establish calibration needs for sensor based systems. Further, it is important for those who make and use sensor based systems to communicate about the sensors themselves. In this study, we employed a newly introduced O_x_ sensor together with a NO_2_ sensor that included an ozone filter. The results from this work may be the first to show results from this combination of cells with high quality NO_2_ measurement performance. Interest in the expanded use of low-cost sensors has been growing among researchers and the citizen scientist, but actual experience with multiple gas monitoring systems is still limited. There is an urgent need to more fully characterize these systems and the sensors used to sense ambient pollutants. This study is one of the first to include the evaluation of multiple important pollutant constituents and their data correction in a challenging Asian ambient environment.

## Figures and Tables

**Table 1:** Regression results of the sensor V_Ref_ with temperature and relative humidity (all *p* < 0.001).

Sensor	CO-B4	NO-B4	NO_2_-B4	O_X_-B4
R^2^	0.35	0.94	0.56	0.45
T-weight (m V/°C)	−1.28	3.16	−1.15	−0.38
RH-weight (m V/%)	1.12	0.03	−0.70	−0.67

**Table 2:** Gas interference of sensors.

Gas	Sensor				
	CO-B4	NO-B4	NO_2_-B4	O_x_-B4
CO @1 ppm	NA	<1%	<1%	<1%
NO @100 ppb	<1%	NA	<1%	<1%
NO_2_ @100 ppb	<1%	<1%	NA	100%
O_3_ @100 ppb	<1%	<1%	<1%	NA

**Table 3:** Statistics of regression between sensor and reference equipment data by differential.

Sensor	CO-B4				NO-B4				NO_2_-B4				O_3_-B4			
	Calibration		Validation		Calibration		Validation		Calibration		Validation		Calibration		Validation	
	1σ	R^2^	1σ	Mean	1σ	R^2^	1σ	Mean	1σ	R^2^	1σ	Mean	1σ	R^2^	1σ	Mean
	(ppm)		(ppm)	(ppm)	(ppb)		(ppb)	(ppb)	(ppb)		(ppb)	(ppb)	(ppb)		(ppb)	(ppb)
**Model 0**	0.03	0.96	0.050	0.06	6.4	0.82	11.6	−9.2	6.5	0.84	6.6	−2.6	5.8	0.70	4.5	12.7
**Model 1**	0.02	0.98	0.046	−0.01	5.4	0.87	7.8	−5.5	5.8	0.79	6.5	−1.2	5.4	0.73	6.1	7.9
**Model 2**	0.03	0.97	0.061	0.01	5.3	0.87	8.7	−3.2	7.5	0.87	15.9	−15.2	5.5	0.73	13.4	7.2
**Model 3**	0.02	0.98	0.057	0.01	5.4	0.87	8.9	1.4	5.8	0.87	7.0	−2.3	5.6	0.72	7.0	2.7
